# Circulating Tumor Biomarkers in Meningiomas Reveal a Signature of Equilibrium Between Tumor Growth and Immune Modulation

**DOI:** 10.3389/fonc.2019.01031

**Published:** 2019-10-10

**Authors:** Erdogan Pekcan Erkan, Thomas Ströbel, Christian Dorfer, Markus Sonntagbauer, Andreas Weinhäusel, Nurten Saydam, Okay Saydam

**Affiliations:** ^1^Research Program in Systems Oncology, Faculty of Medicine, University of Helsinki, Helsinki, Finland; ^2^Institute of Neurology, Medical University of Vienna, Vienna, Austria; ^3^Department of Neurosurgery, Medical University of Vienna, Vienna, Austria; ^4^Austrian Institute of Technology, Molecular Diagnostics Center for Health and Bioresources, Vienna, Austria; ^5^Department of Biochemistry, Molecular Biology, and Biophysics, Medical School, University of Minnesota, Minneapolis, MN, United States; ^6^Division of Hematology and Oncology, Department of Pediatrics, Medical School, University of Minnesota, Minneapolis, MN, United States

**Keywords:** meningioma, proximity extension assay, biomarker, serum biomarker, CNS tumors, high-throughput immunoassay cancer panel

## Abstract

Meningiomas are primary central nervous system (CNS) tumors that originate from the arachnoid cells of the meninges. Recurrence occurs in higher grade meningiomas and a small subset of Grade I meningiomas with benign histology. Currently, there are no established circulating tumor markers which can be used for diagnostic and prognostic purposes in a non-invasive way for meningiomas. Here, we aimed to identify potential biomarkers of meningioma in patient sera. For this purpose, we collected preoperative (*n* = 30) serum samples from the meningioma patients classified as Grade I (*n* = 23), Grade II (*n* = 4), or Grade III (*n* = 3). We used a high-throughput, multiplex immunoassay cancer panel comprising of 92 cancer-related protein biomarkers to explore the serum protein profiles of meningioma patients. We detected 14 differentially expressed proteins in the sera of the Grade I meningioma patients in comparison to the age- and gender-matched control subjects (*n* = 12). Compared to the control group, Grade I meningioma patients showed increased serum levels of amphiregulin (AREG), CCL24, CD69, prolactin, EGF, HB-EGF, caspase-3, and decreased levels of VEGFD, TGF-α, E-Selectin, BAFF, IL-12, CCL9, and GH. For validation studies, we utilized an independent set of meningioma tumor tissue samples (Grade I, *n* = 20; Grade II, *n* = 10; Grade III, *n* = 6), and found that the expressions of amphiregulin and Caspase3 are significantly increased in all grades of meningiomas either at the transcriptional or protein level, respectively. In contrast, the gene expression of VEGF-D was significantly lower in Grade I meningioma tissue samples. Taken together, our study identifies a meningioma-specific protein signature in blood circulation of meningioma patients and highlights the importance of equilibrium between tumor-promoting factors and anti-tumor immunity.

## Introduction

Meningiomas account for 53.1% of all non-malignant brain and other CNS tumors (1). The majority of meningiomas with documented WHO grade is Grade I (80.6%) (1). These benign tumors can remain dormant without causing any symptoms for a long time, which arguably represents the major challenge in early detection of meningiomas (2). Intriguingly, recurrence frequently occurs in tumors with benign histology, and recent studies revealed that coexistence of del(1p36) and monosomy 14 is associated with early recurrence of meningiomas (3). Mutations or deletions on the *NF2* gene, which is located on 22q12.2 locus and encodes Merlin, have been originally described in meningiomas as an oncogenic driver gene (4). However, recent studies showed that other genetic alterations in *TRAF7, PIK3CA, KLF4, POLR2A AKT1, SMO, SUFU*, and *SMARCB1* genes are involved in meningioma pathogenesis (5–8). Grade II and III meningiomas are also associated with few specific recurrent somatic mutations, such as SMARCE1 mutations in clear cell meningioma and BAP1 mutation in a subset of rhabdoid meningiomas (9). Treatment protocol for meningomas is closely associated with tumor location, grade and includes surgery followed by fractionated external beam radiation therapy (EBRT) (10).

To date, no consensus has been established on specific biomarkers toward early diagnosis or prognosis for meningiomas. Most CNS tumors are currently diagnosed primarily radiology-based modalities like CT or MRI scans followed by validation with genetic or IHC-based diagnostic markers. The major challenge in the radiology-based technology is that the tumors can be detected only when they reach to a certain size, which creates worse prognostic risk as tumor is transformed from benign to malignant forms.

While CT and MRI are usually sufficient for diagnosis of meningiomas, other tumors and diseases may radiologically mimic meningioma and complicates the diagnosis (11). In addition, imaging modalities are only able to detect tumors when they reach a certain size. Given the slow growth rate of meningiomas, these tumors may remain undiagnosed for extended periods of time. Grade I meningiomas have a mean tumor age of >20 years, highlighting requirement of longer time periods to diagnose tumor. Furthermore, the average time between initial cell transformation and detection of tumor mass has been reported as 26.3 years in fibrous meningiomas, and 17.8 years in meningothelial meningiomas (12). Slow growth rate of meningiomas also complicates early prediction of the meningioma progression, and recurrence that occurs in ~30% of Grade I meningiomas, 50% of Grade II and 80% of Grade III meningiomas (13, 14).

Currently, there is no serum-based diagnostic and/or prognostic marker available to monitor transition stages of meningiomas from benign state to malignant form. Identification of such markers would not only improve early detection of meningioma, but also improve survival rate of meningioma patients.

Proteomics analysis has been used to investigate disease pathophysiology and identify potential surrogate disease markers for brain tumors (15–18). However, only a small number of reports focused on protein profiling of meningioma tumor specimens (19, 20) and serum samples (21). The majority of proteomics studies employed meningioma tumor tissues (22–25), while others used biological fluids, such as cerebrospinal fluid and serum (21).

In this study, for the search of potential biomarkers for meningiomas, we used a high-throughput, multiplex immunoassay cancer panel based on the proximity extension assay (PEA) to screen a set of 92 cancer-related protein markers. The serum protein expression profiles of Grade I (benign, *n* = 23), Grade II (atypical, *n* = 4), and Grade III (anaplastic, *n* = 3) meningioma patients were analyzed in relation to the healthy control subjects (*n* = 12). Furthermore, our validation studies using an independent set of meningioma tumor tissues (Grade I, *n* = 20; Grade II, *n* = 10; and Grade III, *n* = 6) identifies a protein biomarker signature in meningioma patient sera.

## Materials and Methods

### Study Population

The screening cohort consisted of Grade I (*n* = 23), Grade II (*n* = 4), and Grade III (*n* = 3) meningioma patients. The independent validation cohort consisted of Grade I (*n* = 20), Grade II (*n* = 10), Grade III (*n* = 6) meningioma patient tissue samples that were collected after the surgery and stored at −80°C. Patients were operated at the Vienna General Hospital (Vienna, Austria). This study was carried out in accordance with the Good Scientific Practice recommendations of the ethics committee of Medical University of Vienna. All subjects gave written informed consent in accordance with the Declaration of Helsinki. The protocol was approved by the local ethics committee of the Medical University of Vienna.

### Serum Collection

Preoperative blood samples were collected before any therapeutic intervention (surgery, chemotherapy, radiotherapy). Blood samples were let to stand at 4°C for 60 min, and then centrifuged at 1,100 × g for 10 min. Serum samples were aliquoted and stored at −80°C.

### Protein Detection With Proximity Extension Assay (PEA)

Serum samples from Grade I (*n* = 23), Grade II (*n* = 4), and Grade III (*n* = 3) meningioma patients were delivered to AIT Molecular Diagnostics. Age- and gender-matched control serum samples (*n* = 12) were provided by AIT Molecular Diagnostics. OLINK ProSeek Oncology-I Panel was used to detect expression of 92 cancer-related proteins (https://www.olink.com/products/oncology) (Supplementary Table 1). Briefly, 1 μL serum samples were incubated with 92 antibody pairs of oligonucleotide-labeled antibodies. Binding of the antibody pair to the antigen brings the oligonucleotides to close proximity, and a new PCR target sequence is formed by a proximity-dependent DNA polymerization event. The resulting sequence is subsequently detected and quantified by high-throughput real-time PCR (BioMarkTM HD System, Fluidigm Corporation). The fluorescent signals generated in real-time PCR directly correlates with protein abundance. Raw Cq values were normalized by subtracting the Cq values for the extension control and compared to that of the corresponding background reaction. The resulting ddCq values were used for further analysis and represented as Normalized Protein Expression (NPE) in Log2 scale.

### Functional Analysis

Differentially expressed proteins (FDR = 0.01) identified in Reproducibility-optimized test statistic (ROTS) were analyzed with the PANTHER database version 14.0 (http://www.pantherdb.org/) (26). The list of proteins was uploaded and mapped against the reference dataset (*Homo sapiens*).

### Real Time-qPCR

High-Capacity cDNA Reverse Transcription Kit (Applied Biosystems) was used to reverse transcribe 1,000 ng of total RNA. Quantitative real-time PCR reactions were performed using the Universal PCR Master Mix (Thermo Scientific) on a 7300 Real-Time PCR system (Applied Biosystems). ΔCt values were calculated according to the following formula (27): ΔCt = Ct_*Target*_ – CT_*Reference*._ This equation considers Ct values to be proportional to the negative logarithm of gene expression. Thus, ΔCt values are positively related to the expression of gene of interest. Glyceraldehyde 3-phosphate dehydrogenase (GAPDH) was used as a reference gene to normalize gene expression. Primer sequences are shown in Supplementary Table 3.

### Western Blotting

Western blotting was performed as previously described (28). The following primary antibodies were used: Caspase-3 (1:2,000, BD Biosciences) and beta-actin (1:5,000, Sigma-Aldrich). Densitometric analysis was performed on scanned blot images. Images were transformed to gray-scale on ImageJ software (v.1.50i, National Institutes of Health, USA). For each blot, a lane normalization factor was calculated by dividing the signal of each loading control band with that of the highest signal of loading control on the blot. The calculated lane normalization factor was then used to normalize caspase 3 band signals.

### Statistical Analysis

Reproducibility-optimized test statistic (ROTS) algorithm (26) was used to identify differentially expressed proteins. Comparisons were done between Grade I preoperative samples and control samples. The default parameters (*B* = 1,000, *K* = 19) were used for all comparisons. Proteins with a false discovery rate (FDR) below 0.01 were considered as significant.

RStudio version 1.1.414 and Prism version 8.0.1 (GraphPad Inc.) were used for statistical analysis. Multiple tests (Anderson-Darling, D'Agostino and Pearson, Shapiro-Wilk, and Kolmogorov-Smirnov tests) were used to test for log-normal distribution. For proteins that followed normal distribution, paired *t-*test was used to compare protein expression between the samples. For proteins that did not follow normal distribution, Wilcoxon matched-pairs signed rank test was used. Brown-Forsythe ANOVA test with Dunnett's multiple comparison was used to compare mRNA expression levels among meningioma grades. *P* < 0.05 were considered statistically significant.

## Results

### Serum Protein Profiling With a High-Throughput, Multiplex Immunoassay Cancer Panel Using the Proximity Extension Assay

To identify protein biomarkers associated with meningioma, we used a high-throughput, multiplex immunoassay cancer panel consisting of 92 putative cancer-related human proteins that are involved in key biological processes, such as angiogenesis, cell-to-cell signaling, cell-cycle control, and inflammation which play central roles in cancer metabolism. The putative protein biomarkers that are selected based on the analyses of commonly used bioinformatic databases [e.g., Uniprot, Human Protein Atlas, Gene Ontology (GO), and DisGeNET] are then classified according to their functional protein groups, roles in biological processes, associations with diseases, and expression patterns in tissues (Olink Proteomics) (Supplementary Table 1). The multiplex nature of this immunoassay-based cancer panel enables simultaneous analysis of large sample numbers, and its coupling with the PEA technology provides uncompromised data quality. Thus, taking advantage of this technology, we analyzed 30 serum samples obtained from meningioma patients prior to the surgery, and 12 control serum samples from the age- and gender-matched healthy subjects.

**Figure 1:**
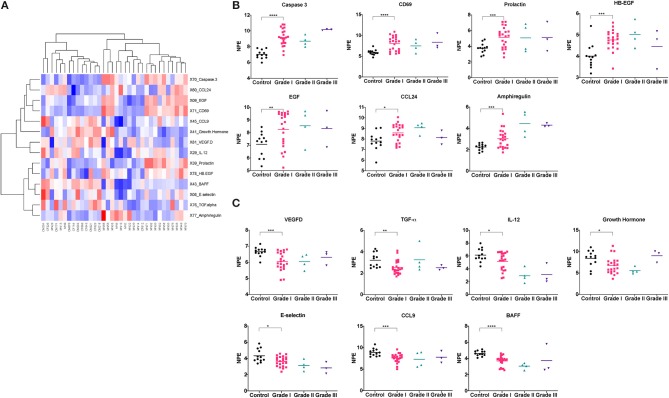
Differentially expressed proteins between meningioma patients and control subjects. ROTS algorithm was used to identify differentially expressed proteins between Grade I meningioma patients and healthy control subjects **(A)**. Heatmap visualization of differentially expressed proteins **(B,C)**. Comparison of protein levels across tumor grades (*P < 0.05, **P < 0.01, ***P < 0.001, ****P < 0.0001).

Our results showed that 14 proteins were differentially expressed in the Grade I meningioma patients compared to healthy control subjects (Figure 1). Of those, seven proteins, caspase-3, CD69, prolactin, epidermal growth factor (EGF), chemokine (C-C) ligand 24 (CCL24), amphiregulin (AREG), and heparin-binding EGF (HB-EGF) were highly expressed in the Grade I meningioma samples (Figure 1B), while, the other seven proteins, vascular endothelial growth factor D (VEGF-D), transforming growth factor alpha (TGF-α), E-selectin, B-cell activating factor (BAFF), interleukin-12 (IL-12), chemokine (C-C motif) ligand 9 (CCL9), and Growth Hormone were reduced in the meningioma serum samples, compared to healthy control subjects (Figure 1C and Table 1). The PANTHER pathway analyses revealed that differentially expressed proteins are linked to the EGF receptor signaling (EGF, amphiregulin, HB-EGF, TGF-α), apoptosis (caspase-3), immunomodulation (prolactin, CD69, CCL24, IL-12, CCL9, BAFF), and angiogenesis (VEGF-D). Of note, the expression levels of 16 analytes were below the limit of detection in more than 20% of all samples. These markers were excluded from the downstream analyses (Supplementary Table 2).

**Table 1:** Differentially expressed proteins between Grade I meningioma patients and control subjects.

**Protein**	**log2FC (Meningioma/ Control)**	**Linear FC (Meningioma/ Control)**	***P*-value**	**FDR**
Caspase-3	2.13	4.38	0	0.00
CD69	2.09	4.27	3.29E-05	0.00
Prolactin	1.37	2.58	4.47E-04	0.00
EGF	1.18	2.26	2.89E-03	0.00
CCL24	0.79	1.72	8.68E-03	0.00
Amphiregulin	0.78	1.72	5.66E-03	0.00
HB-EGF	0.76	1.69	2.16E-03	0.00
VEGFD	−0.61	0.66	8.64E-03	0.00
TGF-α	−0.67	0.63	9.88E-03	0.00
E-selectin	−0.69	0.62	9.55E-03	0.00
BAFF	−0.93	0.53	4.54E-04	0.00
IL-12	−1.00	0.50	8.41E-03	0.00
CCL9	−1.33	0.40	2.89E-04	0.00
Growth Hormone	−1.66	0.32	1.82E-03	0.00

*FC, fold change; FDR, false discovery rate*.

We further questioned whether the differentially expressed proteins detected in the blood circulation of the meningioma patients were correlated with the tumor grade. Although the patterns of the protein profiles of the Grade II and Grade III patients were comparable to that of the Grade I patients, we were unable to statistically analyze the differences between these groups due to the small sample sizes of Grade II (*n* = 4) and Grade III (*n* = 3) patients.

### Validation Cohort Using an Independent Set of Meningioma Tumor Tissues

**Figure 2:**
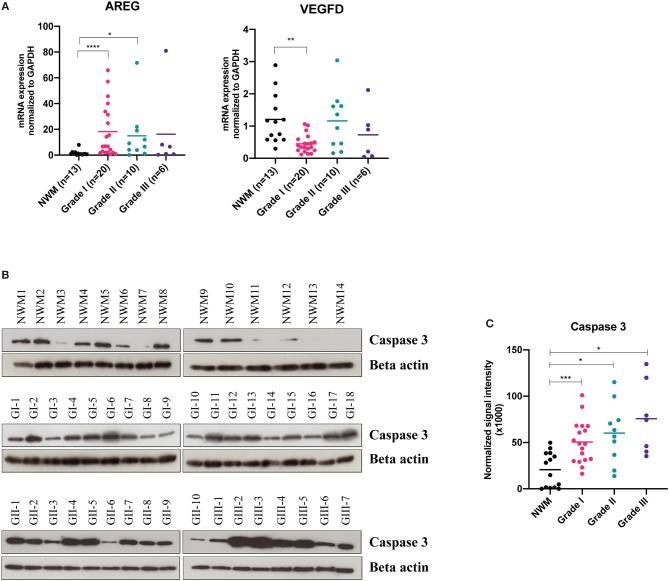
Validation studies of cancer-panel protein screening candidates. For validation studies, by using an independent meningioma tissue specimen set, *n* = 36 [Grade I (*n* = 20), Grade II (*n* = 10), and Grade III (*n* = 6)], the expressions of the indicated genes were analyzed by RT-qPCR, Normal white matter (NWM) tissue samples were used as control (AREG, NWM vs. Grade I, ****P < 0.0001, Brown-Forsythe ANOVA test with Dunnett's multiple comparison; VEGF-D, NWM vs. Grade I, **P < 0.01, Brown-Forsythe ANOVA test with Dunnett's multiple comparison) **(A)**. Western blot images showing caspase-3 protein expression in tumor lysates obtained from Grade I (*n* = 18), Grade II (*n* = 10), and Grade III (*n* = 7) meningiomas **(B)**. Densitometric analysis of Western blot bands (NWM vs. Grade I, ***P < 0.001, Brown-Forsythe ANOVA test with Dunnett's multiple comparison; NWM vs. Grade II, and NWM vs. Grade III, *P < 0.05, Brown-Forsythe ANOVA test with Dunnett's multiple comparison) **(C)**.

For validation studies, we enrolled an independent set of 49 meningioma tissue samples including Grade I, *n* = 20; Grade II, *n* = 10; grade III, *n* = 6, as well as Normal White Matter (NWM), *n* = 13 as control tissue samples. As shown in Figure 2A, we found a significant increase in the gene expression levels of amphiregulin in the Grade I meningiomas, while the VEGF-D mRNA levels were relatively decreased, compared to the control NWM tissue samples. In addition, we analyzed the protein expression levels of Caspase 3 enzyme in the tissue samples, and found that the protein levels of Caspase 3 are significantly higher in all grades of meningioma tissue samples compared to the control group (Figures 2B,C). We did not observe significant difference in the gene expression profiles of CD69, BAFF, HB-EGF, E-Selectin (Supplementary Figure 1) suggesting that these proteins may not exclusively originate from meningioma tumor cells, but rather may be secreted from other cell types, such as immune system cells that circulate in the bloodstream.

## Discussion

Identification of non-invasive protein biomarkers has been a great interest for cancer diagnosis. In this study, we searched for putative cancer biomarkers that can be used for diagnostic and prognostic purposes in meningioma patients. For this discovery study, we employed a commercial cancer panel based on a high-throughput, multiplex immunoassay that is equipped with the PEA technology, and detected serum protein profiles of the meningioma patients (*n* = 30) in comparison to the control subjects (*n* = 12). By taking advantage of the technology of PEA that requires low sample input to carry out multiplexed assays with good sensitivity and specificity (29, 30), we identified 14 differentially expressed proteins in the sera of the Grade I meningioma patients relative to the healthy control subjects (Figures 1A–C). In addition, the proteins differentially expressed in the Grade I meningioma patient sera were also detected in a small set of Grade II (*n* = 4) and Grade III (*n* = 3) patient sera (Figures 1A,B). However, due to the small sample size of higher grade meningioma patient samples, we were unable to determine how significantly protein expressions differed between the tumor grades. In order to validate our potential biomarkers, we utilized an independent cohort containing 36 meningioma tumor samples, and found that amphiregulin and Caspase 3 are significantly increased in meningioma tumor tissues, while VEGF-D is relatively lower in comparison to the control NWM tissue samples (Figure 2). The other candidate proteins, Prolactin, HB-EGF, E-Selectin, CD69, and BAFF that were emerged from the cancer-panel protein screening were not detected in our independent set of meningioma tissue specimens (Supplementary Figure 1).

In our study, caspase-3 emerged as the top differentially expressed protein showing increased expression levels in our dataset of the meningioma patient sera (*n* = 30). Caspase-3 is a central effector of apoptosis (31). However, sublethal activation of caspase-3 has been shown to promote genetic instability and carcinogenesis (32). In addition, caspase-3 was proposed to play a key role in the repopulation of tumors in response to radiotherapy, and its activity is correlated with increased rate of recurrence and death in cancer patients (33). Our findings showing increased serum levels of caspase-3 are supported by an earlier study reporting an increased caspase-3 immunoreactivity in meningioma tissues, in which Grade II and Grade III meningioma tissues exhibited higher scores of immunopositivity relative to the benign Grade I meningioma tissues (34). In addition, the same study identified caspase-3 as an independent predictor of early recurrence (34). Elevated serum caspase-3 levels in meningioma patients, along with its reported increased tissue levels, may indicate a clinical value for caspase-3 in meningioma as a predictive marker of benign-to-malignant transformation. In line with our study, a recent study has also shown that increased caspase-3 expression in primary atypical and malignant meningiomas is correlated with the higher grade of meningioma (35).

CD69, an early activation marker of lymphocytes and natural killer cells (36) is another hit detected in our screening study. CD69 is an important regulator of immune responses that take part in cytokine release, homing and migration of lymphocytes (37, 38). However, the state of tissue environment and cytokine spectrum could differentially regulate its role in immune responses (39). In murine models, CD69 deficiency is associated with enhanced anti-tumor immunity and longer survival (40). Recently, CD69 has been indicated in the induction of T cell exhaustion in a breast cancer tumor model in mice, where anti-CD69 antibody treatment was proven to enhance anti-tumor activity, pointing CD69 as a novel target for cancer immunotherapy (41). On the contrary, high levels of tumor-infiltrating CD4^+^CD69^+^ T cells are associated with good prognosis in head and neck squamous cell carcinoma (42). In meningioma, an increased infiltration of CD69^+^ lymphocytes, along with tissue macrophages and natural killer cells has been reported, which is specifically associated with the cases carrying a distinct cytogenetic profile of isolated monosomy 22/del(22q) that shows a better prognosis (43). Thus, increased infiltration of meningiomas by activated lymphocytes including CD69^+^ subsets may be associated with immune surveillance, and elimination of tumor cells that restricts the tumor growth (43). Given that our study identified CD69 as a putative serum biomarker in meningiomas, it is plausible to think that this may be caused by the increased presence of tumor growth-limiting CD69^+^ lymphocytes in the circulation of Grade I meningioma patients. However, further studies are required to investigate the presence of CD69^+^ lymphocytes in the blood stream of the meningioma patients and its correlation with serum CD69 protein for possible clinical applications.

Our study identified two different ligands of EGFR, amphiregulin (AREG) and heparin-binding EGF (HB-EGF) that are elevated in the sera of Grade I meningioma patients. Among those, our validation studies showed that the expression of amphiregulin is significantly upregulated in the meningioma tumor tissues, compared to the control NWM specimens. Amphiregulin is a secreted EGFR ligand that regulates cellular growth and differentiation, immunity, inflammation, and tissue repair (44, 45), and it is overexpressed in a variety of cancers (44, 46, 47). Amphiregulin is originally described as an epithelial- and mesenchymal cell-derived factor, however, recent studies show that it is expressed by multiple populations of activated immune cells including dendritic cells and CD4^+^ T cells (45). It is likely that elevated levels of amphiregulin in meningioma patient sera may reflect the response of immune system and/or the ongoing tissue repair owing to the tumor mass. Alternatively, amphiregulin and HB-EGF may act as activators of EGFR that is overexpressed in meningiomas, supporting tumor growth and malignancy (48).

Prolactin was found in elevated levels in the sera of our Grade I meningioma patient group in our study. However, we did not detect an increase in prolactin levels in meningioma tissues. Interestingly, some increase in prolactin receptor levels in meningiomas has been also reported earlier (49, 50). Considering that prolactin stimulates growth of primary meningioma cells *in vitro* (51), further studies are necessary to reveal the clinical importance of serum prolactin levels in meningioma patients.

CCL24 is a chemokine with well-studied roles in allergies (52). In the context of cancer, CCL24 expression has been shown to associate with poor prognosis in colorectal cancer and also contribute to hepatocellular carcinoma malignancy via the RhoB-VEGFA-VEGFR2 angiogenesis pathway (53, 54). In our study, we detected slightly higher serum levels of CCL24 in Grade I meningioma patients in comparison to the control subjects. However, clinical value of this finding is yet to be understood in meningiomas.

IL-12 is a cytokine that is mainly produced by antigen-presenting cells, and it has well-established anti-tumor activity (55, 56). IL-12 serum levels were found relatively reduced in meningioma and glioblastoma patients (57). Consistently, our study revealed that Grade I meningioma patients have reduced serum IL-12 levels compared to the control subjects.

VEGF plays a pivotal role in angiogenesis, and its expression in meningiomas was reported to associate with unfavorable prognosis and recurrence (58–60). Intriguingly, several studies addressing serum VEGF levels in meningioma depicted some contradictory results. Stockhammer et al. reported that serum VEGF-A concentration is higher in patients with central nervous system tumors, including meningioma, than in patients with no tumor diagnosis (61). Nowacka et al. reported higher serum VEGF-A levels in meningioma patients (62). In our study, we detected that the serum levels of VEGF-D, a member of the VEGF family that plays a role in glioblastoma angiogenesis (63), is relatively lower in the Grade I meningioma patients compared to the control subjects. Furthermore, we showed that the gene expression of VEGF-D is significantly lower in meningioma tumor tissues relative to the control NWM tissues. In line with our findings, another group reported lower serum levels of VEGF in meningioma patients where control subjects had higher levels of serum VEGF (64). Although the exact mechanism of lower serum and tissue levels of VEGF in meningioma is unknown, further research is required if VEGF levels could have some prognostic value in following up tumor recurrence.

B-cell activating factor (BAFF), a member of the TNF superfamily is one of the critical factors controlling B-cell homeostasis, and its interaction with its ligand/receptor regulates the survival and proliferation of malignant cells (65, 66). High BAFF serum levels have been detected in autoimmune disorders, including rheumatoid arthritis and systemic lupus erythematosus, as well as in the malignancies, such as non-Hodgkin's lymphoma, B-CLL, and multiple myeloma (65). Interestingly, BAFF is expressed in astrocytes and astrocyte-derived BAFF promotes B-cell survival in multiple sclerosis and primary CNS lymphoma (67). Our findings revealing low levels of circulating BAFF in Grade I meningioma might indicate an autocrine control of the BAFF system that can counteract the malignant progression of meningiomas.

Similarly, we observed that the patients with Grade I meningioma had lower levels of serum E-selectin, compared to the healthy control subjects. Given the roles of selectins in cancer progression and metastasis (68), low levels of E-selectin might reflect the benign state of meningiomas.

Chemokines and their receptors play important roles in determining the metastatic destination of cancers (69). Chemokine CCL9 (MIP-1γ) is a ligand for the CCR1/CD191 receptor present on T cells, monocytes, macrophages, some myeloid-derived suppressor cells, and osteoclasts (70), and it is a potent chemo-attractant for immune cells (71). Moreover, CCL9/CCR1 signaling has been shown to recruit myeloid progenitors to tumor area, leading to progression of adenomas to carcinomas and also enhancing tumor invasion (70, 72–74). CCL9 has also been suggested as a fair candidate for anti-metastasis treatment of cancer (75). In our study, circulating CCL9 appeared to be lower in Grade I meningioma relative to the control group, in a similar way to the serum levels of VEGF, BAFF, E-selectin, and IL-12. It is conceivable to postulate that low serum levels of CCL9, VEGF, BAFF, E-selectin, and IL-12 might reflect the benign state of the disease that may be used for monitoring the tumor progression from benign to malignant form.

In sum, this study provides a list of candidate proteins that could potentially be utilized as diagnostic/prognostic circulating biomarkers of meningioma. Our validation studies in an independent set of meningioma tissue specimens provide further evidence that caspase-3, amphiregulin and VEFG-D might be promising markers to monitor the status of meningiomas through a non-invasive manner by using patient blood.

## Data Availability Statement

The raw data supporting the conclusions of this manuscript will be made available by the authors, without undue reservation, to any qualified researcher.

## Ethics Statement

Patients were operated at the Vienna General Hospital (Vienna, Austria). This study was carried out in accordance with the Good Scientific Practice recommendations of the ethics committee of Medical University of Vienna. All subjects gave written informed consent in accordance with the Declaration of Helsinki. The protocol was approved by the local ethics committee of the Medical University of Vienna.

## Author Contributions

OS and NS conceived and designed the study. CD provided the blood samples. EE isolated the serum samples. AW and MS conducted the serum analysis. TS performed the RT-qPCR and WB experiments. EE and TS analyzed the data. EE, NS, and OS wrote the manuscript. All authors read and approved the final version of the manuscript.

### Conflict of Interest

The authors declare that the research was conducted in the absence of any commercial or financial relationships that could be construed as a potential conflict of interest.
