# Supplementary material for: Circulating Tumor Biomarkers in Meningiomas Reveal a Signature of Equilibrium Between Tumor Growth and Immune Modulation
Source: Front Oncol. 2019 Oct 10;9:1031. doi: 10.3389/fonc.2019.01031 (PMC6795693; doi:10.3389/fonc.2019.01031)
Supplement: Supplementary file 1 [file Data_Sheet_1.PDF]

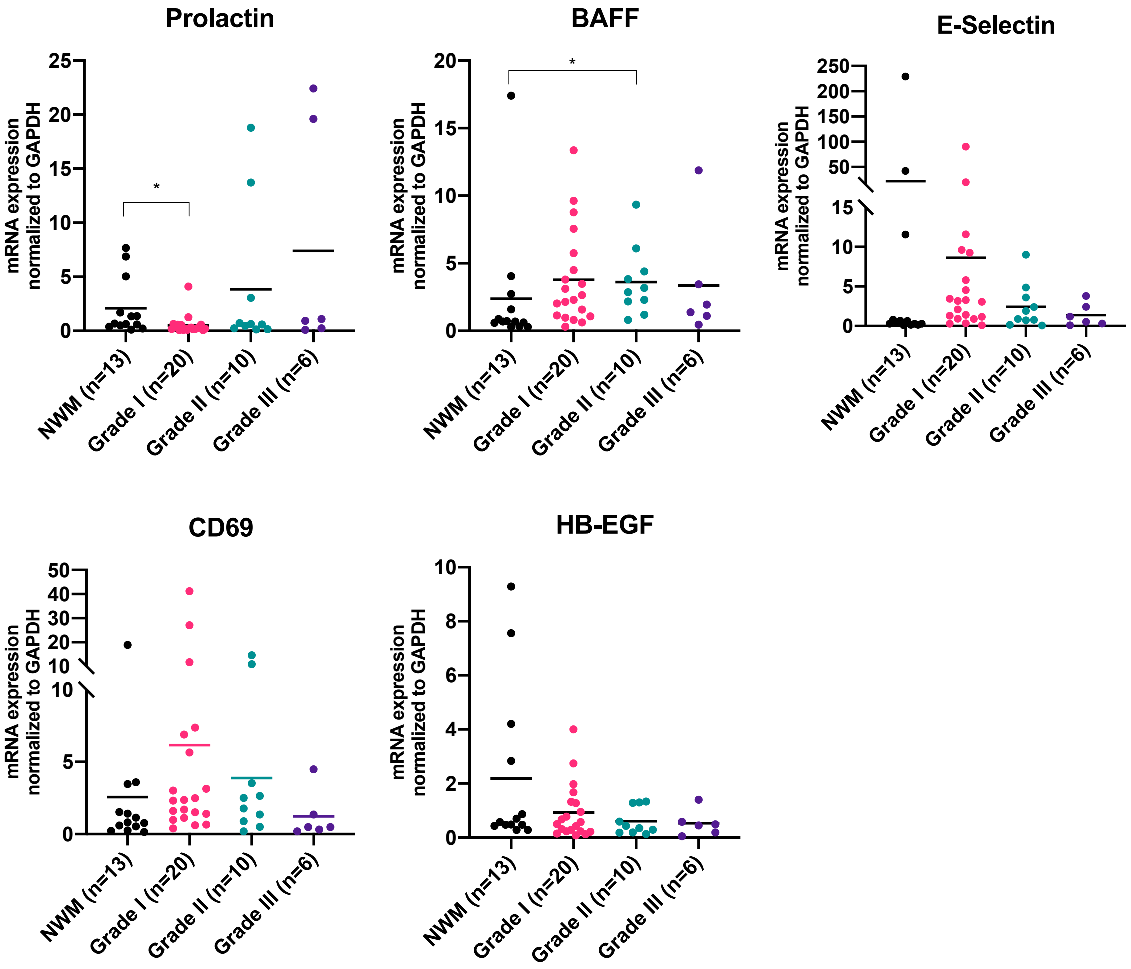

SI Figure 1 We used RT-qPCR to analyze the mRNA expression of differentially expressed proteins in an independent validation cohort. In contrast with the screening results, prolactin and HB-EGF mRNA expression was lower in Grade I meningioma tumor tissues, whereas BAFF mRNA expression was higher in all grades. E-Selectin and CD69 mRNA expression matched the screening results, but the differences in mRNA expression levels were not statistically significant (Brown-Forsythe ANOVA test with Dunnett's multiple comparison, $p>0.05$)

SI Table 1   Protein markers included in the ProSeek Oncology-I Panel

01_IL-8	51_CXCL10
02_VEGF-A	52_Ep-CAM
03_Adrenomedullin	53_ErbB2/Her2
05_CD40 ligand	54_ErbB3/Her3
06_GDF-15	55_ErbB4/Her4
07_PlGF	56_HGF
08_E-selectin	57_PSA
09_EGF	58_MYD88
10_Osteoprotegerin	59_MIA
11_IFN-gamma	60_CCL24
12_IL-1ra	61_Midkine
13_IL-6	62_U-PAR
14_Cystatin B	63_CXCL5
15_MCP-1	64_Cathepsin D
16_Kallikrein-6	65_Betacellulin
17_Galectin-3	66_Epiregulin
18_EPO	67_Flt3L
20_LAP TGF-beta-1	68_VEGFR-2
21_Kallikrein-11	69_CCL21
22_TIE2	70_Caspase-3
23_Tissue Factor	71_CD69
24_TNF-RI	72_TNFRSF4
25_PDGF subunit B	73_TR-AP
26_GM-CSF	74_CD30-L
27_CSF-1	75_REG-4
28_CXCL11	76_TGF-alpha
29_IL-12	77_Amphiregulin
30_IL-2	78_HB-EGF
31_IL-7	79_MIC-A
32_Stem cell factor	80_IL-4
33_CXCL9	81_VEGF-D
34_IL6RA	82_HE4
35_TNF-R2	83_CXCL13
36_MMP-3	84_EGFR
37_IL2RA	85_HGF receptor
38_TNFSF14	86_Thrombopoietin
39_Prolactin	87_FABP4
40_MPO	88_CEA
41_Growth Hormone	89_TNF
42_FasL	90_CA242
43_BAFF	91_CA-125
44_FAS	92_Prostasin
45_CCL9	93_Follistatin
48_Estrogen receptor	94_PECAM-1
49_EMMPRIN	95_IL17RB
50_CAIX	96_FR-alpha

SI Table 2 Protein markers excluded from analyses

Protein	Number of samples at LOD	Percentage of samples at LOD
**11_IFN-gamma**	43	60
**18_EPO**	23	32
**26_GM-CSF**	72	100
**30_IL-2**	72	100
**36_MMP-3**	26	36
**48_Estrogen receptor**	72	100
**57_PSA**	40	56
**58_MYD88**	18	25
**65_Betacellulin**	71	99
**66_Epiregulin**	26	36
**80_IL-4**	41	57
**88_CEA**	52	72
**89_TNF**	57	79
**90_CA242**	70	97
**91_CA-125**	30	42
	**Number of samples with negative expression value**	**Percentage of samples with negative expression value**
**79_MIC-A**	64	88

SI Table 3 Primers used in RT-qPCR experiments

Target gene	Strand	Sequence
**GAPDH**		
	Forward	ACATCGCTCAGACACCATG
	Reverse	TGTAGTTGAGGTCAATGAAGGG
**HB-EGF**		
	Forward	GATCTGGACCTTTTGAGAGTCA
	Reverse	TGCAGAAGTCCTTGTATTTCCG
**AR**		
	Forward	GCTGTCGCTCTTGATACTCG
	Reverse	CTTCCCAGAGTAGGTGTCATTG
**CD69**		
	Forward	ACATGGTGCTACTCTTGCTG
	Reverse	CTTTGCCATTTGACCACTTCC
**VEGFD**		
	Forward	AATTAGTGCCTGTTAAAGTTGCC
	Reverse	AGGACAGAGTTTCTTGGAATGG
**E-Selectin**		
	Forward	TTGCAAGTGTGACCCTGG
	Reverse	TGTAGCTGAAGTTTCCCAGTG
**Prolactin**		
	Forward	AACCAAACGGCTTCTAGAGG
	Reverse	ATAAGCAGAAAGGCGAGACTC
**BAFF**		
	Forward	ACGCCATGGGACATCTAATTC
	Reverse	TTCCAGTTTTGCAATGCCAG
